# Supplementary material for: Evaluation of statistical methods used in the analysis of interrupted time series studies: a simulation study
Source: BMC Med Res Methodol. 2021 Aug 28;21:181. doi: 10.1186/s12874-021-01364-0 (PMC8403376; doi:10.1186/s12874-021-01364-0)
Supplement: Supplementary file 1 — Additional file 1: Appendices This file contains the appendices referenced in the text. STurner_ITS_Simulation_Appendices.docx [file 12874_2021_1364_MOESM1_ESM.docx]

Appendices

Study:

“Evaluation of statistical methods used in the analysis of interrupted time series studies: a simulation study”

Authors:

Simon L Turner^1^, Andrew B Forbes^1^, Amalia Karahalios^1^, Monica Taljaard^2,3^, Joanne E McKenzie^1^

Affiliations:

^1^School of Public Health and Preventive Medicine, Monash University, 533 St Kilda Road, Melbourne, Victoria, Australia.

^2^Clinical Epidemiology Program, Ottawa Hospital Research Institute, Ottawa, Ontario, Canada. 1053 Carling Ave, Ottawa.

^3^School of Epidemiology, Public Health and Preventive Medicine, University of Ottawa, Ottawa, Ontario, Canada. 75 Laurier Ave E, Ottawa.

1. Statistical method details
   1. Ordinary Least Squares

Model (1) can be written in a matrix form as:

| *Y* = *X*β + ε | *(3)* |
| --- | --- |

where *Y* and ε are *n × 1* vectors whose *t^th^* element is *y_t_* and *ε_t_* respectively, *X* is the *n × 4* design matrix with $t^{'}th row \left( 1, t, D_{t}, D_{t}I\left( t-T_{1} \right) \right)$, and $\epsilon_{t}\sim N(0, \sigma^{2})$. The OLS estimator of $\beta$ is $\hat{\beta}_{OLS}=\left( X'X \right)^{-1}X'Y$, and ${Var(\hat{\beta}}_{OLS})={\sigma^{2}\left( X'X \right)}^{-1}$.

- 1. Newey West

The NW estimator (lag-1) of $\beta$ is just the OLS estimator, $\hat{\beta}_{NW}=\hat{\beta}_{OLS}$, but with a sandwich variance estimator of the form

| $\hat{Var}\left( \hat{\beta}_{NW} \right)=\left( X'X \right)^{-1}X'\hat{\Omega}X\left( X'X \right)^{-1}$ | *(5)* |
| --- | --- |

where:

| $X^{'}\hat{\Omega}X=X^{'}\hat{\Omega}_{0}X+\frac{n}{n-k}\frac{1}{2}\sum_{t=2}^{n} \hat{e}_{t}\hat{e}_{t-1}\left( x_{t}^{'}x_{t-1}+x_{t-1}^{'}x_{t} \right)$ | | *(6)* |
| --- | --- | --- |
| $X^{'}\hat{\Omega}_{0}X=\frac{n}{n-k}\sum_{i} \hat{e}_{i}^{2}x_{i}^{'}x_{i}$ | *(7)* | |
| $\hat{e}_{i}=y_{i}-x_{i}\hat{\beta}_{OLS}$ | *(8)* | |

where *X* is the same $n\times4$ design matrix as specified for OLS above. The central term in the variance expression allows for empirical determination of autocorrelation and heteroskedasticity (1).

- 1. Generalised Least Squares

In the Cochrane-Orcutt and Prais-Winsten methods, from the equations (1) and (2), the dependent and independent variables are transformed to create a new model in which the error terms are uncorrelated:

| $Y_{t}^{*}=Y_{t}-\rho Y_{t-1}$ | *(9a)* |
| --- | --- |
| $X_{t}^{*}=X_{t}-\rho X_{t-1}$ | *(9b)* |

Then fit $Y_{t}^{*}=X_{t}^{*}\beta+w_{t}$, where

| ${w_{t}=\varepsilon}_{t}-\rho\varepsilon_{t-1}\sim N(0, \sigma^{2})$ | *(10)* |
| --- | --- |

using OLS, and iterate until convergence.

Generally, the correlation is unknown, and must first be estimated. An estimate of autocorrelation at each iteration can be obtained using the OLS residuals $e_{t}$ from fitting Equation (2) as above:

| $\hat{\rho}=\frac{\sum_{t=2}^{n} e_{t-1}e_{t}}{\sum_{t=2}^{n} e_{t-1}^{2}}$ | *(11)* |
| --- | --- |

The CO method discards the first observation, while the PW method retains the first observation, but applies the following transformation (2):

| ${y_{1}^{*}=\sqrt{1-\rho^{2}}y}_{1} and X_{1}^{*}=\sqrt{1-\rho^{2}}X_{1}$, where $X_{1}$ is the first row of X. | *(12)* |
| --- | --- |

- 1. ARIMA/ARMAX Regression with Autoregressive errors estimated using maximum likelihood

The ARIMA model may include information from previous time points. In an ARIMA model with first order autocorrelation only, i.e. ARIMA(1,0,0), equations (1) and (2) are fit simultaneously by maximum likelihood (1). ARMAX models add covariates to ARIMA models (1, 3).

- 1. Durbin-Watson test for autocorrelation

The Durbin-Watson test statistic is given by:

| $D=\frac{\sum_{t=2}^{n} \left( e_{t}-e_{t-1} \right)^{2}}{\sum_{t=1}^{n} e_{t}^{2}}$ | *(13)* |
| --- | --- |

For test statistic values under two, D is compared to lower ($d_{L})$ and upper ($d_{U})$bounds, leading to either a conclusive or inconclusive result. For test statistic values over two, 4-D is compared to the lower and upper bounds and a conclusive $H_{alternative}$indicates the presence of negative autocorrelation:

| $If D>d_{U}, conclude H_{o}$ |  |
| --- | --- |
| $If D<d_{L}, conclude H_{alternative}$ |  |
| $If d_{L}\leq D\leq d_{U}, inconclusive$ |  |

Lower ($d_{L})$and upper ($d_{U}$) bounds can be found in tables online or in textbooks, e.g. Kutner et al (2008)(4).

1. Definitions of performance measures

The definitions of performance measures used to compare statistical methods are given in Table 1.

Table 1: Definitions of performance measures. Where $\theta$ represents the parameter under investigation, $\hat{\theta}$ being the estimate of that parameter, $\bar{\theta}$ being the mean value of the estimate, $n_{sim}$ being the number of simulations (in this study, 10,000), $p_{i}$ being the p-value of estimate $i$ and $\alpha$ being the significance level (5).

| Performance measure | Definition | Estimate |
| --- | --- | --- |
| Bias | $E\left[ \hat{\theta} \right]-\theta$ | $\frac{1}{n_{sim}}\sum_{i=1}^{n_{sim}} \hat{\theta}_{i}-\theta$ |
| Empirical standard error | $\sqrt{Var\left( \hat{\theta} \right)}$ | $\sqrt{\frac{1}{n_{sim}-1}\sum_{i=1}^{n_{sim}} \left( \hat{\theta}_{i}-\bar{\theta} \right)^{2}}$ |
| Mean square error | $E\left[ \left( \hat{\theta}_{i}-\theta\right)^{2} \right]$ | $\frac{1}{n_{sim}}\sum_{i=1}^{n_{sim}} \left( \hat{\theta}_{i}-\theta\right)^{2}$ |
| Coverage | $Pr\left( \hat{\theta}_{low}\leq\theta\leq\hat{\theta}_{upp} \right)$ | $\frac{1}{n_{sim}}\sum_{i=1}^{n_{sim}} 1\left( \hat{\theta}_{low,i}\leq\theta\leq\hat{\theta}_{upp,i} \right)$ |
| Power | $Pr\left( p_{i}\leq\alpha\right)$ | $\frac{1}{n_{sim}}\sum_{i=1}^{n_{sim}} 1\left( p_{i}\leq\alpha\right)$ |

1. References

1. StataCorp. Stata 15 Base Reference Manual. College Station, TX: Stata Press; 2017.

2. Prais SJ, Winsten, C.B. Trend estimators and serial correlation. In: University Y, editor. Cowles Commision1954.

3. Paolella MS. Linear models and time-series analysis : regression, ANOVA, ARMA and GARCH: Hoboken, NJ : John Wiley & Sons, Inc.; 2019.

4. Kutner M, Nachtscheim C, Neter J, Li W, Senter H. Applied linear statistical models. In: Kutner M, Nachtscheim C, Neter J, Li W, Senter H, editors. 2008. p. 880-.

5. Morris TP, White IR, Crowther MJ. Using simulation studies to evaluate statistical methods. Statistics in Medicine. 2019;38(11):2074-102.
